# SCREENING FOR VISION AND HEARING LOSS IN OLDER ADULTS WITH DEMENTIA: A FEASIBILITY STUDY

**DOI:** 10.1093/geroni/igz038.442

**Published:** 2019-11-08

**Authors:** Katherine McGilton, Fiona Höbler, Marilyn Reed, Tammy Labreche, M Kathleen Pichora-Fuller, Valerie Shoemaker, Walter Wittich

**Affiliations:** 1 KITE, Toronto Rehab, University Health Network, Toronto, Ontario, Canada; 2 Baycrest Health Sciences, Toronto, Ontario, Canada; 3 School of Optometry and Vision Science, University of Waterloo, Waterloo, Ontario, Canada; 4 Department of Psychology, University of Toronto, Toronto, Ontario, Canada; 5 School of Optometry - Université de Montréal, Montréal, Québec, Canada

## Abstract

Sensory loss accounts for one of the most common chronic conditions among older adults, with hearing loss affecting half of adults aged over 65 years and vision loss almost one fifth of those aged 70 years and over. Together, dual sensory loss is found to be most prevalent in older adults with dementia. The highest prevalence is found in long-term care (LTC) settings. For this reason, we conducted a multi-stage study to identify the most effective vision and hearing screening tools for use with older adults living with dementia and to evaluate their feasibility of use by nurses working in LTC. We first conducted a comprehensive review of the literature, and supplemented this with an environmental scan of healthcare professionals and sensory specialists working with older adults who have dementia. Following this extensive review and consultative decision-making process, a package of vision and hearing screening tools was selected for use by nurses working in LTC. On-site training was provided by two experienced audiologists and optometrists, after which the feasibility of sensory screening by three nurses of 17 residents under their care was evaluated. We report on the six measures of hearing and seven measures of vision that were piloted for screening of older adults with dementia living in LTC, and on the findings for their feasibility of use by nurses working in this setting. Recommendations regarding the feasibility and reliability of screening for vision and hearing loss in older adults with dementia are discussed.

